# The significance of investigation into underlying coronary artery fistula in patients with tricuspid valve endocarditis: a case report

**DOI:** 10.1093/ehjcr/ytaf260

**Published:** 2025-05-22

**Authors:** Yoshiro Tanaka, Jun Yoshida, Yusuke Kashiwagi, Takashi Kunihara, Michihiro Yoshimura

**Affiliations:** Division of Cardiology, Department of Internal Medicine, The Jikei University School of Medicine, 3-25-8, Nishi-Shinbashi, Minato-ku, Tokyo 105-8461, Japan; Division of Cardiology, Department of Internal Medicine, The Jikei University School of Medicine, 3-25-8, Nishi-Shinbashi, Minato-ku, Tokyo 105-8461, Japan; Division of Cardiology, Department of Internal Medicine, The Jikei University School of Medicine, 3-25-8, Nishi-Shinbashi, Minato-ku, Tokyo 105-8461, Japan; Department of Cardiac Surgery, The Jikei University School of Medicine, 3-25-8, Nishi-Shinbashi, Minato-ku, Tokyo 105-8461, Japan; Division of Cardiology, Department of Internal Medicine, The Jikei University School of Medicine, 3-25-8, Nishi-Shinbashi, Minato-ku, Tokyo 105-8461, Japan

**Keywords:** Multimodal imaging, Tricuspid valve infective endocarditis, Congenital heart disease, Coronary artery fistula, Case report

## Abstract

**Background:**

Coronary artery fistula (CAF) is an uncommon congenital heart disease (CHD). Most patients are asymptomatic, and CAFs are typically discovered incidentally on echocardiography or computed tomography coronary angiography (CTCA). Although previous reports have suggested an association between CAF and infective endocarditis, there are few reports on the coexistence of CAF and tricuspid valve infective endocarditis (TVIE). We report a rare case where a CAF was identified as the underlying CHD in TVIE using multimodal imaging. Both the CAF and TVIE were successfully treated surgically.

**Case summary:**

A Japanese woman in her 40 s who presented with a fever of 38°C that persisted for 4 days. *Streptococcus oralis* was isolated from two sets of blood cultures obtained on admission. Tricuspid valve vegetation was detected using transthoracic echocardiography, and a CAF, which originated from the right coronary artery (RCA) and terminated in the right atrium (RA), was detected by CTCA. Furthermore, the CAF and turbulent shunt flow directed towards the tricuspid valve were identified on transoesophageal echocardiography and subsequently confirmed as an RCA–RA fistula on coronary angiography. After antibiotic therapy, the patient underwent vegetation resection, tricuspid valve annuloplasty, and CAF excision.

**Discussion:**

Careful investigation of the underlying CHD is necessary when TVIE is found. In patients with TVIE, multimodal imaging is useful for investigating underlying CAF and the direct association between TVIE and CAF. When multimodal imaging suggests a direct association between CAF and TVIE, surgical resection of both the TVIE and CAF is essential to prevent the recurrence of TVIE.

**Learning points**

- A careful investigation with multimodal imaging into underlying congenital heart disease (CHD), including coronary artery fistula (CAF), is necessary when tricuspid valve infective endocarditis (TVIE) is found.
- Surgical resection of CAF may be essential to prevent recurrent TVIE when CAF is considered to be associated with TVIE.

## Introduction

The risk factors for right-sided infective endocarditis include congenital heart disease (CHD), indwelling catheters, cardiovascular electronic implanted devices, and intravenous drug use.^1^ Most right-sided infective endocarditis cases involve the tricuspid valve.^2^ Unrepaired ventricular septal defects have been reported to be a risk factor for tricuspid valve infective endocarditis (TVIE)^3^ in CHD; however, there are currently few reports on the association between TVIE and other types of CHD.

Coronary artery fistulas (CAFs) are a rare form of CHD with abnormal communication between the coronary artery and the cardiac chamber or great vessel.^4^ Coronary artery fistulas are asymptomatic and are coincidentally diagnosed by cardiac imaging, such as echocardiography, computed tomography coronary angiography (CTCA), and coronary angiography.^5^ Although previous reports have shown that CAF is complicated by infective endocarditis,^6^ there are few reports of CAF-associated TVIE. We report a rare case of TVIE in which multimodal imaging demonstrated the presence of CAF and the direct association between TVIE and CAF.

## Summary figure

**Figure:**
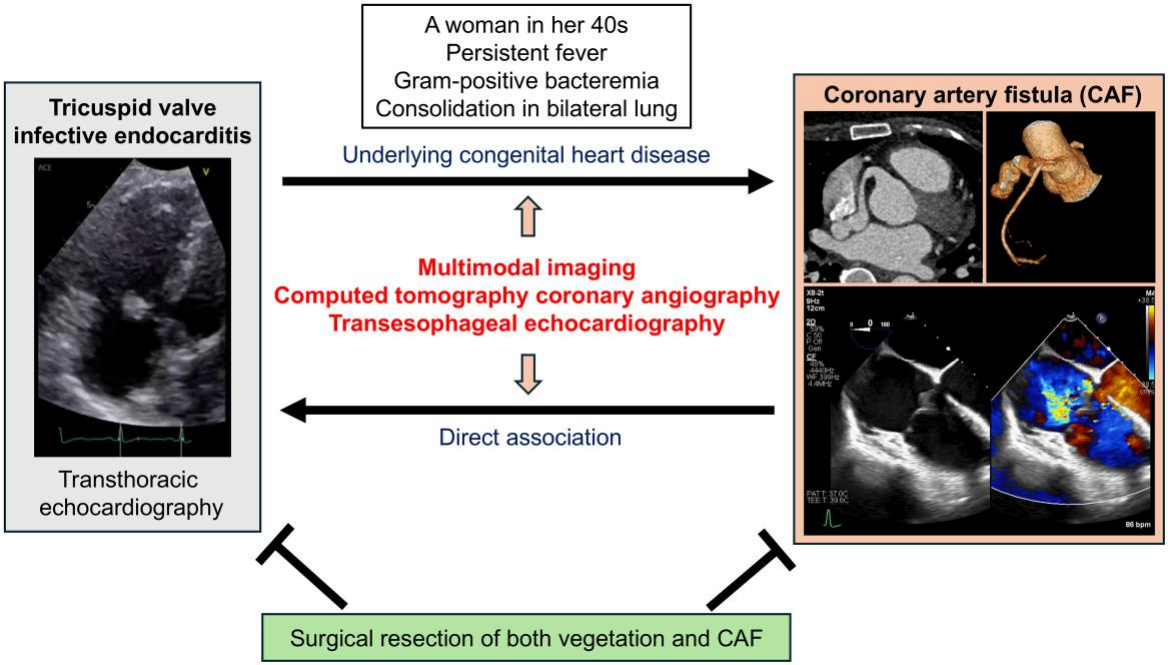


## Case report

**Figure 1:**
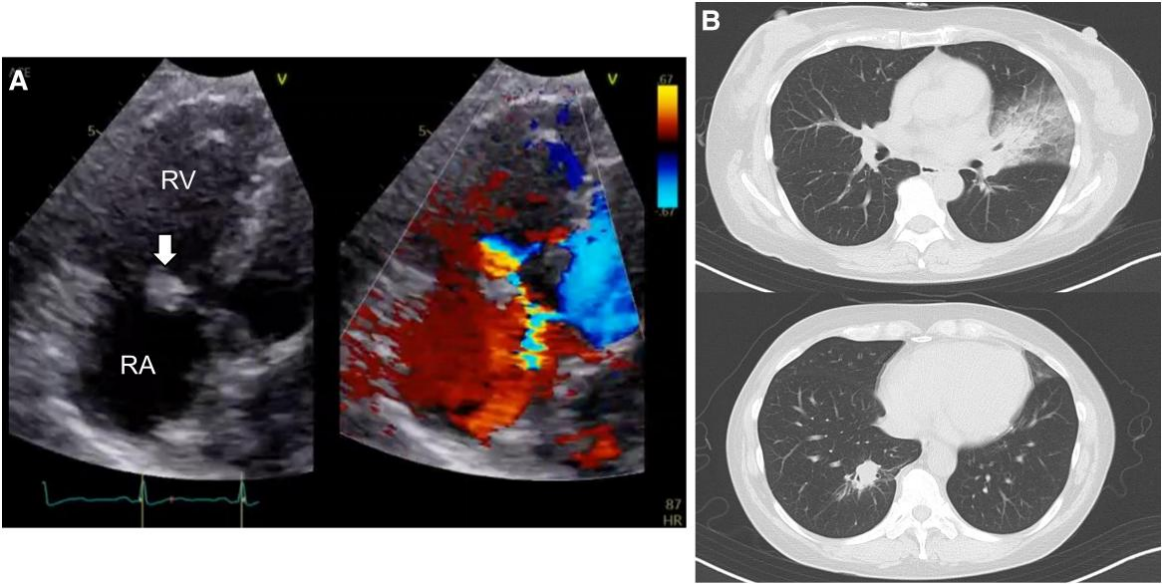
Chest computed tomography and transthoracic echocardiography indicated septic embolism in the lung due to tricuspid valvular infective endocarditis. (*A*) Transthoracic echocardiography showed vegetation (Thin arrow) was attached to the tricuspid valve concomitant with mild to moderate tricuspid valve regurgitation. (*B*) Chest computed tomography showed bilateral consolidation in the lingular segment of the left lung and the right lower lobe. RA, right atrium; RV, right ventricle.

A Japanese woman in her 40 s was admitted to our hospital because of a fever exceeding 38°C that persisted for 4 days. She was not an intravenous drug user and had no history of haemodialysis. The patient had a medical history of atopic dermatitis since childhood and had been treated with topical steroids. Additionally, she was recently diagnosed with left-sided breast cancer and had undergone partial mastectomy 2 weeks prior. Chest computed tomography (CT) revealed consolidation in the lower right lung, and the patient was diagnosed with pneumonia. Intravenous ampicillin–sulbactam was administered; however, her fever did not improve, and *Streptococcus oralis* was isolated from two sets of blood cultures obtained on admission. Since the minimum inhibitory concentration of penicillin-G for *S. oralis* was 0.5 µg/mL and that of ampicillin was 1.0 µg/mL, the *S. oralis* was diagnosed with reduced susceptibility to penicillin. Therefore, the antibiotic therapy was changed from ampicillin–sulbactam to ceftriaxone on Day 5. Transthoracic echocardiography (TTE) was performed on Day 8 to detect infective endocarditis. Transthoracic echocardiography showed a large vegetation (15.5 × 10 mm) attached to the tricuspid valve, concurrent with mild to moderate tricuspid valve regurgitation (*Figure 1A*). Furthermore, chest CT showed new pulmonary consolidation in the left lung and residual consolidation in the right lung, suggesting septic embolism due to TVIE (*Figure 1B*). Contrast-enhanced CT and brain magnetic resonance imaging revealed no embolisms in the body, other than in the lungs. According to the modified Duke criteria (*Table 1*),^1^ the patient fulfilled two major criteria [typical microorganisms (*S. oralis*) consistent with two sets of blood cultures and an echocardiogram positive for infective endocarditis] and two minor criteria [fever defined as temperature >38°C and embolic vascular dissemination (pulmonary septic emboli)]. Therefore, the patient was diagnosed with infective endocarditis. As the fever remained over 38.0°C and C-reactive protein remained elevated, the fever was suspected to be associated with ceftriaxone-induced drug fever. The antibiotic regimen was changed from ceftriaxone to meropenem combined with gentamicin on Day 9, with the expectation of synergistic effects. The patient was transferred to our department on Day 12.

**Table 1:** Definition of infective endocarditis according to the modified Duke criteria

**Major criteria**
(i) Blood cultures positive for infective endocarditis. (a) Typical microorganisms consistent with infective endocarditis from two separate blood cultures: Oral streptococci, *Streptococcus bovis*, HACEK group, *Staphylococcus aureus*, *Enterococcus faecalis*. (b) Microorganisms consistent with infective endocarditis from continuously positive blood cultures: • At least two positive cultures of blood samples drawn >12 h apart; or • All of 3 or a majority of ≥4 separate blood cultures (with first and last samples drawn ≥1 h apart). (c) Single positive blood culture for *Coxiella burnetii* or Phase I IgG antibody titre >1:800.
(ii) Imaging positive for infective endocarditis: Valvular, perivalvular/periprosthetic and foreign material anatomic and metabolic lesions characteristic of infective endocarditis detected by any of the following imaging techniques: • Echocardiography, CTCA, [18F]-FDG-PET/CT, or WBC SPECT/CT.
**Minor criteria**
• Predisposing conditions (i.e. predisposing heart condition or people who inject drug use). • Fever defined as temperature >38°C. • Embolic vascular dissemination (including those asymptomatic detected by imaging only): Major systemic and pulmonary emboli/infarcts and abscesses/haematogenous osteoarticular septic complications (i.e. spondylodiscitis)/mycotic aneurysms/intracranial ischaemic and haemorrhagic lesions/conjunctival haemorrhages, and Janeway’s lesions. • Immunological phenomena: glomerulonephritis, Osler nodes, Roth spots, and Rheumatoid factor. • Microbiological evidence: positive blood culture but does not meet a major criterion as noted above or serological evidence of active infection with organism consistent with infective endocarditis.
**Infective endocarditis Classification (at admission and during follow-up)**
Definite: • Two major criteria. • One major criterion and at least three minor criteria. • Five minor criteria. Possible: • One major criterion and one or two minor criteria. • Three–four minor criteria. Rejected: • Does not meet criteria for definite or possible at admission with or without a firm alternative diagnosis.

[18F]-FDG-PET/CT, 18F-fluorodeoxyglucose positron emission tomography; CTCA, computed tomography coronary angiography; SPECT, single photon emission computed tomography; HACEK, *Haemophilus*, *Aggregatibacter*, *Cardiobacterium*, *Eikenella*, and *Kingella*; Ig, immunoglobulin; WBC, white blood cell.

The vital signs (parameters) at the time of transfer were as follows: body temperature, 38.0°C; blood pressure, 90/60 mmHg; pulse rate, 90 beats/min; respiration rate, 16 breaths/min; and peripheral oxygen saturation, 97% on room air. A physical examination revealed left chest pain during breathing and no obvious peripheral oedema. No apparent erythematous or haemorrhagic macular lesions were observed on the palms and soles, and no petechial haemorrhages were noted on the palpebral conjunctiva. Electrocardiography on admission showed sinus rhythm with supraventricular premature contractions. No significant ST-segment changes were observed. Blood tests on transfer to our department showed elevation of the following parameters: white blood cell count [15 × 10^9^/L (reference range 3.3–8.6 × 10^9^); neutrophils, 83.0%; lymphocytes, 9.3%], C-reactive protein [290 mg/L (reference range < 1.4 mg/L)], aspartate aminotransferase [106 IU/L (reference range 13–30 IU/L)], alanine aminotransferase [118 IU/L (reference range 7–23 IU/L)], brain natriuretic peptide (39.9 pg/mL (reference range < 18.4 pg/mL), and troponin I [0.054 ng/mL (reference range < 0.024 ng/mL)]. Evaluation of the patient’s coagulation parameters revealed a prolonged prothrombin time-international normalized ratio of 1.3, activated partial thromboplastin time of 69.8 s (reference range 24–39 s) and elevated D-dimer [2.1 μg/mL (reference range < 1.0 μg/mL)]. Blood revealed a normal creatinine of 0.53 mg/dL [estimated glomerular filtration rate 95 mL/min/1.73 m^2^ (reference range > 60 mL/min/1.73 m^2^)]. After the administration of meropenem and gentamicin for 4 days, the antibiotics were further switched to a combination of cefotaxime and gentamicin owing to the elevation of transaminase levels on Day 12. The two sets of blood cultures obtained on Day 6 were negative on Day 12.

**Figure 2:**
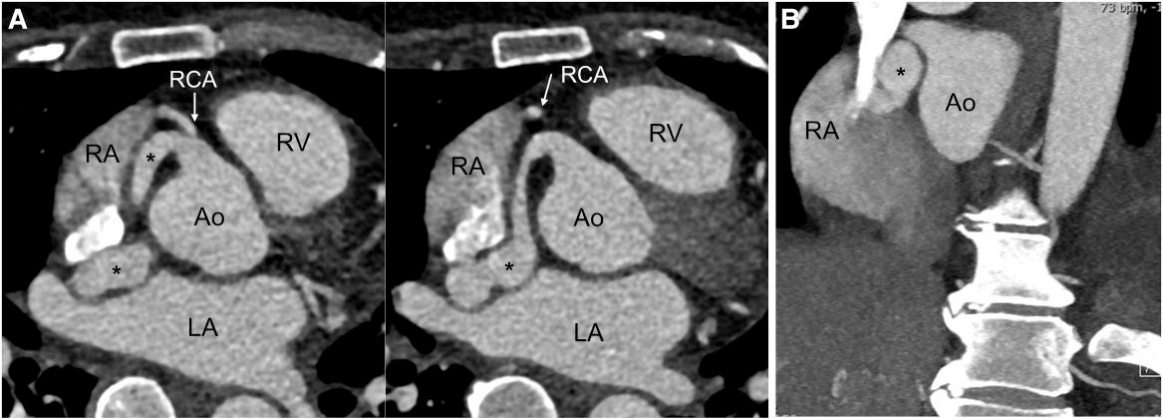
Computed tomography coronary angiography showed a coronary artery fistula from the RCA to the right atrium. (*A*) Axial oblique slices on computed tomography, (*B*) coronal view showing coronary artery fistula (asterisk) draining into the right atrium. RA, right atrium; RV, right ventricle; Ao, aortic root; LA, left atrium; RCA, right coronary artery.

**Figure 3:**
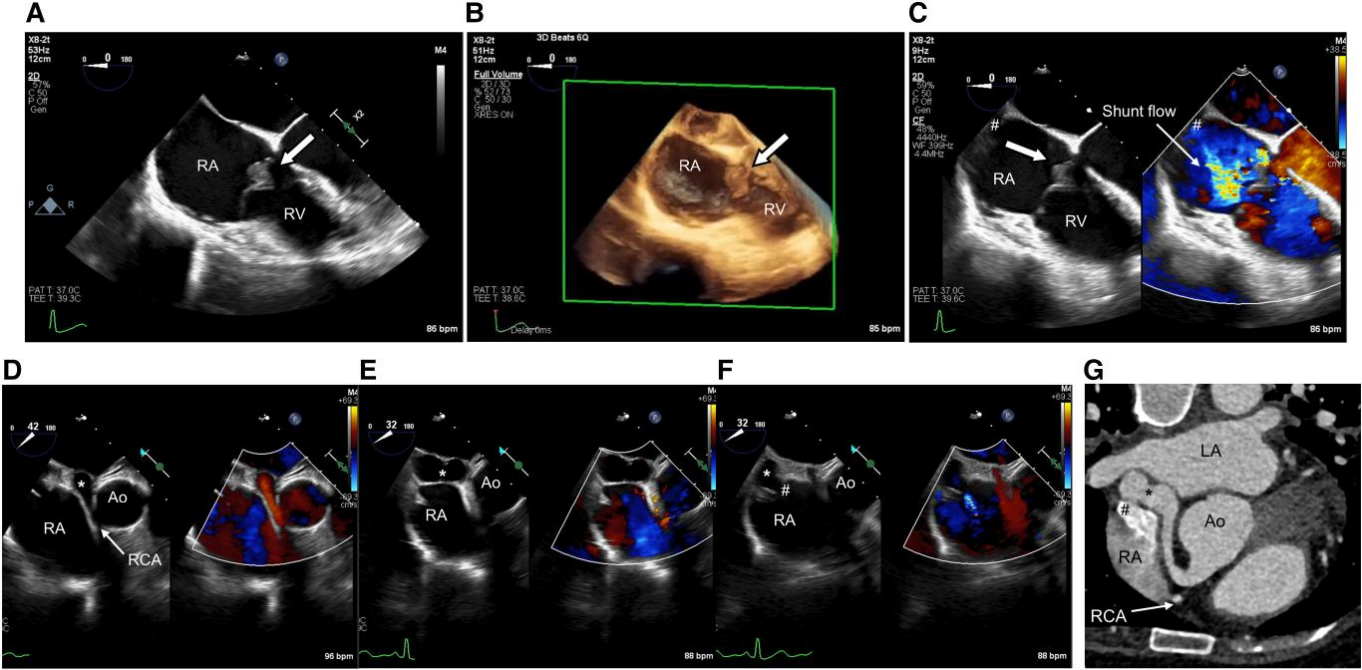
Transoesophageal echocardiography demonstrates vegetation attaching to the tricuspid valve and turbulent shunt flow from coronary artery fistula to the tricuspid valve. (*A, B*) Transoesophageal echocardiography and 3D transoesophageal echocardiography showed the vegetation attaching to the anterior leaflet of the tricuspid valve. (*C*) Transoesophageal echocardiography showed turbulent shunt flow towards the tricuspid valve from the outlet of the fistula to the RA (number sign). (*D*) Transoesophageal echocardiography showed coronary artery fistula (asterisk) originating from the right coronary artery, (*E*) running around the right atrium, and (*F*) terminating at the right atrium. The number sign shows the outlet of the fistula to the right atrium. (*G*) An axial oblique slice on computed tomography coronary angiography is flipped vertically and horizontally to align with the transoesophageal echocardiography image showing that the coronary artery fistula (asterisk) terminates at the right atrium. The number sign shows the outlet of the fistula to the right atrium. RA, right atrium; RV, right ventricle; Ao, aortic root; LA, left atrium; RCA, right coronary artery.

**Figure 4:**
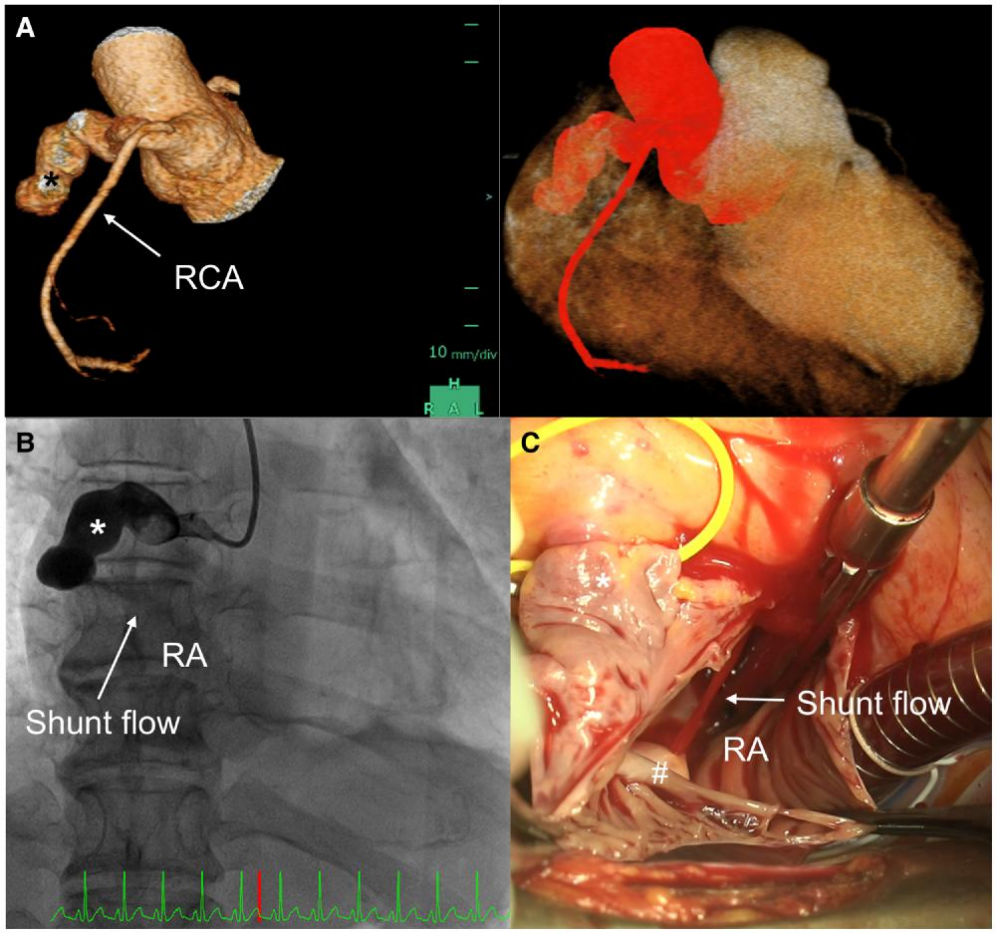
Morphology of the coronary artery fistula and turbulent shunt flow on computed tomography coronary angiography, coronary artery angiography, and a surgical image. (*A*) The morphology of the coronary artery fistula and right coronary artery on 3D computed tomography coronary angiography. (*B*) Coronary angiography showed the coronary artery fistula (asterisk), shunt flow from the coronary artery fistula, and contrast-enhanced right atrium. (*C*) Surgical findings revealed the enlarged coronary artery fistula (asterisk) and shunt flow from the outlet of the fistula (number sign). RA, right atrium; RCA, right coronary artery.

Computed tomography coronary angiography revealed no significant stenosis in the coronary arteries and incidentally detected a dilated fistula from the right coronary artery (RCA) to the right atrium (RA) (*Figures 2*, *3G*, and *4A*) as well as a new pulmonary embolism on chest CT on Day 13. Transoesophageal echocardiography (TEE) revealed vegetation (16 × 10 mm) on the anterior leaflet of the tricuspid valve (*Figure 3A and B*), and continuous turbulent flow from the RA to the tricuspid valve (*Figure 3C*, Supplementary material online, Videos  *S1*  *and S2*). Transoesophageal echocardiography also revealed that the fistula originated from the RCA and terminated in the RA, and an abnormal continuous turbulent jet from the outlet of the fistula moved towards the tricuspid valve leaflet (*Figure 3D–F*). Because recurrent septic pulmonary embolism and CAF, which could be the underlying cause of TVIE confirmed by TEE, were identified, we held a conference with the cardiac surgery team and decided to perform surgical intervention for the resection of both the TVIE and CAF. We also performed coronary angiography and confirmed the presence of a CAF originating from the RCA and terminated the RA (*Figure 4B*), which was also consistent with the findings of computed tomography coronary angiography (CTCA) and TEE.

With the administration of cefotaxime and gentamicin, her fever and inflammatory markers in the blood tests gradually improved, and her C-reactive protein level decreased to 11.2 mg/L by Day 23. On Day 27, we performed surgical intervention for TVIE and CAF. The surgical findings were as follows: vegetation was observed attached to the central tip of the anterior leaflet of the tricuspid valve, and major chordae at that site were found to be ruptured. The vegetation was excised and the attachment site was debrided, allowing the anterior leaflet of the tricuspid valve to be largely preserved. Tricuspid annuloplasty utilizing Tri-Ad Adams tricuspid ring (Medtronic, Minneapolis, MN, USA) and chordoplasty with artificial chordae from the anterior papillary muscle to the anterior tricuspid leaflet were performed. Continuous shunt flow from the outlet of the fistula was detected during surgery (*Figure 4C*). The distal end of the fistula was suture closed at its opening into the RA, and the proximal end was divided just after its origin from the RCA and suture closed. After surgery, chest CT performed on Day 33 (4 weeks after Day 6 when negative blood cultures were confirmed) showed both resolution and progression of consolidation relative to the preoperative findings on Day 13. Therefore, an additional week of antibiotic therapy was administered according to the recommendations of the infectious disease team, and antibiotic therapy was continued until Day 40 (5 weeks after Day 6 when negative blood cultures were confirmed). Neither CTCA nor TTE performed on Day 42 showed any vegetation, and pulmonary consolidation apparently regressed relative to CT on Day 33. After the discontinuation of antibiotic therapy, the patient’s serum inflammatory markers were not elevated, and she was discharged on Day 57. Since then, the patient has remained well for 2 years without any recurrence of infective endocarditis or other cardiovascular events.

## Discussion

This report demonstrates the importance of searching for underlying CHD, such as CAF, when TVIE is found. Multimodal imaging techniques, including CTCA and TEE, are useful not only for identifying the underlying CAF but also for confirming its direct association with TVIE. Surgical resection of the CAF and TVIE may be essential to prevent recurrent CAF-associated TVIE.

Isolated TVIE accounts for only 5%–10% of infective endocarditis.^7^ Infective endocarditis can occur without underlying cardiac diseases, such as in injection drug users. However, most infective endocarditis cases are caused by endothelial injury resulting from abnormal turbulent flow and bacteraemia. Therefore, it is necessary to carefully search for underlying cardiac diseases in which abnormal turbulent flow occurs.

Echocardiography, CTCA, MRI, and coronary angiography are useful diagnostic modalities for CAFs.^8^ In particular, because CTCA involves the whole heart, it can identify the presence of CAFs and allow comprehensive assessment of anatomy, including the specific location, size, and morphology of the fistula.^8^ Infective endocarditis is attributed to turbulent blood flow from shunts, as described previously, and most CAF-associated infective endocarditis cases are valvular infective endocarditis.^6^ A previous report showed that among 25 patients with CAF-associated infective endocarditis, there were three cases of TVIE caused by turbulent shunt flow (fistula characteristics: RCA-coronary sinus, RCA–RA, and RCA-left circumflex artery).^6^ TEE can provide pathophysiological assessment of blood flow with colour Doppler and cardiac structures with moving images. In particular, TEE provided more information about the atrium than TTE in this case. To our knowledge, this is the first study to capture turbulence flow from the RCA–RA fistula towards TVIE, making it possible to confirm the strong association between the two diseases, as demonstrated in *Figure 3*.

Right-sided infective endocarditis generally has a better clinical course than left-sided infective endocarditis, and most patients with right-sided infective endocarditis can be managed with antibiotic therapy without surgery.^1^ Surgery for right-sided infective endocarditis, such as TVIE, is indicated in patients with persistent bacteraemia, right ventricular dysfunction secondary to tricuspid valve regurgitation, respiratory compromise, and recurrent septic pulmonary embolism.^1^ Among these surgical indications, this case corresponds to the criteria for a recurrent septic pulmonary embolism. However, since the inflammatory markers showed an increasing trend with antibiotic treatment, surgical resection of the TVIE would not have been performed, and antibiotic therapy would have been acceptable if there had been no CAF in this case.

The indications for closure of CAF are controversial, and the European Society of Cardiology guidelines for the management of adult CHD do not explicitly state clear surgical indications for CAF disease.^9^ Previous reports have shown that CAF are complicated by arrhythmia, ischaemia, endocarditis, endarteritis, vessel rupture, and ventricular dysfunction.^5^ These complications would prompt consideration of invasive interventions, such as transcatheter closure or surgical correction/ligation of the CAF.^4,5^ Considering the mechanism of CAF-associated infective endocarditis, the remaining CAFs were associated with a high risk of recurrent infective endocarditis, as shown in previous studies.^10–12^ As multimodal imaging strongly suggests an association between CAF and TVIE, it is reasonable that surgical resection of both CAF and TVIE was performed to prevent the recurrence of CAF-associated infective endocarditis in this case.

There are no guidelines regarding whether surgical intervention or catheter-based intervention is preferable for CAF. In 1947, Biork *et al*.^13^ completed the first CAFs surgical closure, and surgical closure of CAFs became the standard choice for closing CAFs because of the low surgical mortality (0%–4%). The first transcatheter closure of CAF was reported in 1983 by Reidy *et al*.^5^ With the advancement of devices such as detachable coils and vascular occluders, along with the minimally invasive nature of catheter-based procedures in comparison to surgery, the number of transcatheter closures has increased. Al-Hijji *et al*.^5^ stated that, in symptomatic cases of CAFs with low surgical risk, particularly when there are additional indications for surgery or when catheter-based intervention is not suitable, it is acceptable to perform surgical ligation of the CAF. Although a case of CAF complicated with infective endocarditis was successfully treated by catheter-based closure of CAF following an adequate course of antibiotic therapy,^14^ performing catheter-based procedures without sufficient antibiotic treatment and introducing foreign materials, such as coils or vascular occluders, generally poses a high risk of recurrence of infective endocarditis. Since recurrent septic pulmonary embolism occurred due to TVIE caused by the RCA–RA fistula and the operative risk was relatively low in this case, surgical intervention was prioritized over catheter-based treatment.

In conclusion, careful investigation of underlying CHD is necessary when encountering patients with TVIE. Multimodal imaging, such as CTCA and echocardiography, are useful for diagnosing CAF-associated infective endocarditis. We believe that surgical resection of both CAF and TVIE is essential to prevent the recurrence of TVIE.

## Supplementary Material

ytaf260_Supplementary_Data

## Data Availability

The data underlying this article will be shared on reasonable to the corresponding author.

## Lead author biography

Dr Yoshiro Tanaka graduated from Tohoku University School of Medicine in 2014 and achieved his PhD degree in 2021 at the Jikei University School of Medicine, Tokyo, where he currently has the position of assistant professor. He is involved in treating patients with cardiovascular diseases while conducting basic and clinical research in cardiovascular metabolism.

## References

[1] Delgado V, Ajmone Marsan N, de Waha S, Bonaros N, Brida M, Burri H, et al 2023 ESC Guidelines for the management of endocarditis. Eur Heart J 2023;44:3948–4042. 37622656 10.1093/eurheartj/ehad193

[2] Akinosoglou K, Apostolakis E, Koutsogiannis N, Leivaditis V, Gogos CA. Right-sided infective endocarditis: surgical management. Eur J Cardiothorac Surg 2012;42:470–479. 22427390 10.1093/ejcts/ezs084

[3] Butensky AM, Channing A, Handel AS, Kalfa D, Holzer S. Tricuspid valve endocarditis in four patients with unrepaired restrictive perimembranous ventricular septal defects. Pediatr Cardiol 2022;43:1929–1933. 35657420 10.1007/s00246-022-02938-5

[4] How WJ, Luckie M, Bratis K, Hasan R, Malik N. Evolving consequences of right coronary artery to right atrium: coronary cameral fistula-a case report. Eur Heart J Case Rep 2024;8:ytae207. 38715625 10.1093/ehjcr/ytae207 PMC11074991

[5] Al-Hijji M, El Sabbagh A, El Hajj S, AlKhouli M, El Sabawi B, Cabalka A, et al Coronary artery fistulas: indications, techniques, outcomes, and complications of transcatheter fistula closure. JACC Cardiovasc Interv 2021;14:1393–1406. 34238550 10.1016/j.jcin.2021.02.044

[6] Said SA . Characteristics of congenital coronary artery fistulas complicated with infective endocarditis: analysis of 25 reported cases. Congenit Heart Dis 2016;11:756–765. 27414233 10.1111/chd.12392

[7] Chan P, Ogilby JD, Segal B. Tricuspid valve endocarditis. Am Heart J 1989;117:1140–1146. 2653011 10.1016/0002-8703(89)90874-0

[8] Zhang W, Maimaitiaili A, Xing Y, Yan F, Huo Q. Case report: surgical repair for left main coronary artery to right atrium fistula with endocarditis. Front Cardiovasc Med 2023;10:1101750. 37123468 10.3389/fcvm.2023.1101750 PMC10130432

[9] Baumgartner H, De Backer J, Babu-Narayan SV, Budts W, Chessa M, Diller GP, et al 2020 ESC Guidelines for the management of adult congenital heart disease. Eur Heart J 2021;42:563–645. 32860028 10.1093/eurheartj/ehaa554

[10] Ong ML . Endocarditis of the tricuspid valve associated with congenital coronary arteriovenous fistula. Br Heart J 1993;70:276–277. 8398501 10.1136/hrt.70.3.276 PMC1025310

[11] Shah K, Jobanputra Y, Sharma P. Recurrent bacteremia in the setting of a coronary artery Fistula. Cureus 2020;12:e9289. 32832287 10.7759/cureus.9289 PMC7437107

[12] Kiel MD, Prathivadhi-Bhayankaram S, Singhal AK, Ashwath ML. A case report of superior vena cava/right coronary artery fistula secondary to chronic endocarditis. Eur Heart J Case Rep 2024;8:ytae240. 38770406 10.1093/ehjcr/ytae240 PMC11104665

[13] Wu YH, Liu YC, Chao MF, Dai ZK, Chen IC, Lo SH, et al Case report: transcatheter closure of a giant and tortuous right coronary artery to right ventricle fistula in an infant. Front Cardiovasc Med 2022;9:898914. 36003905 10.3389/fcvm.2022.898914 PMC9393260

[14] Jariwala U, Hasan RK, Thorn EM, Zakaria S. An unusual case of infective endocarditis involving a right coronary artery to superior vena cava fistula. Catheter Cardiovasc Interv 2015;85:620–624. 25044393 10.1002/ccd.25597

